# Pinpointing the Geographic Origin of 165-Year-Old Human Skeletal Remains Found in Punjab, India: Evidence From Mitochondrial DNA and Stable Isotope Analysis

**DOI:** 10.3389/fgene.2022.813934

**Published:** 2022-04-28

**Authors:** J.S. Sehrawat, Shailesh Agrawal, Deeksha Sankhyan, Monika Singh, Sachin Kumar, Satya Prakash, Richa Rajpal, Gyaneshwer Chaubey, Kumarasamy Thangaraj, Niraj Rai

**Affiliations:** ^1^ Department of Anthropology, Panjab University, Chandigarh, India; ^2^ Birbal Sahni Institute of Palaeosciences, Lucknow, India; ^3^ Cytogenetic Laboratory, Department of Zoology, Banaras Hindu University, Varanasi, India; ^4^ CSIR-Centre for Cellular and Molecular Biology, Hyderabad, India; ^5^ Centre for DNA Fingerprinting and Diagnostics, Hyderabad, India

**Keywords:** ancient DNA, stable isotope, forensic, anthropology, mtDNA, human population genetics

## Abstract

In 2014, 157 years after the Sepoy Mutiny of 1857, several unidentified human skeletons were discovered in an abandoned well at Ajnala, Punjab. The most prevailing hypothesis suggested them as Indian soldiers who mutinied during the Indian uprising of 1857. However, there is an intense debate on their geographic affinity. Therefore, to pinpoint their area of origin, we have successfully isolated DNA from cementum-rich material of 50 good-quality random teeth samples and analyzed mtDNA haplogroups. In addition to that, we analyzed 85 individuals for oxygen isotopes (δ^18^O values). The mtDNA haplogroup distribution and clustering pattern rejected the local ancestry and indicated their genetic link with the populations living east of Punjab. In addition, the oxygen isotope analysis (δ^18^O values) from archaeological skeletal remains corroborated the molecular data and suggested the closest possible geographical affinity of these skeletal remains toward the eastern part of India, largely covering the Gangetic plain region. The data generated from this study are expected to expand our understanding of the ancestry and population affinity of martyr soldiers.

## 1 Introduction

Mitochondrial DNA (mtDNA) has provided forensic scientists a valuable tool for determining the genetic origin of damaged, degraded, or very limited biological samples. Due to the low DNA recovery from ancient remains, mtDNA is always the best choice to target as sequences from multi-copy loci have more chance to be detected in ancient remains than the single-copy sequences due to their presence in larger numbers per cell (Merheb et al., 2019). Furthermore, mtDNA is widely used to trace matrilineal ancestry as it passes from the mother to the offspring.

The nuclear DNA is either unavailable or highly degraded and insufficient in most forensic situations where only severely putrefied tissues, degraded bones, or teeth are available for identification purposes. The mtDNA analysis is the only option left with forensic anthropologists to attempt identification of such challenged human remains retrieved from forensic or bioarchaeological scenarios (Budowle et al., 2003; Rai et al., 2014; Chaubey et al., 2017; Harney et al., 2019). Each cell contains several hundreds and thousands of copies of mtDNA compared to only two copies of autosomal DNA, so the chances of recovery of mtDNA from badly damaged human osseous remains of forensic interest are increased manyfold (Holland et al., 1993; Lutz et al., 1996). Recent advances in ancient DNA (aDNA) analyses, facilitated by improved sequencing technologies, have helped the experts glean information about past human populations’ identity, genetic history, migration patterns, and admixtures (Haber et al., 2016; Shinde et al., 2019).

The survival and recovery of DNA from human hard tissues like bones and teeth has become an essential scientific tool in forensic anthropological investigations (Adler et al., 2011). Little is known about the quality and quantity of mtDNA extracted from the preserved teeth or bones, so there is a pressing need within forensic anthropology to conduct molecular studies on the human skeletal remains preserved at different stages in diverse conditions. Teeth and bones are morphologically and biochemically different (Dobberstein et al., 2008). No potential differences have been reported in the ancient DNA content within different dental tissues or regions (Shiroma et al., 2004; Alakoc and Aka, 2009). It is a small size and mineralized status of the tooth, which imparts toughness and reduced porosity to the dental enamel and cementum to ensure the safer protection of dental DNA from various environmental challenges and contaminations (Rudbeck et al., 2005; Sampietro et al., 2006; Adler et al., 2011). Tooth DNA is generally of higher quality and less prone to decomposition or decay than bone DNA (Ricaut et al., 2005; Gilbert et al., 2005). Molecular analysis of teeth samples has proven their reliability and suitability for molecular identification of unknown human remains recovered from disaster sites, mass graves, or crime scenes (Rohland and Hofreiter, 2007).

The physical methods of tooth powdering have potential influences on the quantity and quality of DNA extracted from it. Out of two commonly used powdering techniques (pulverizing or drilling), direct drilling of the tooth with a dental burr or Dremel bit is the most preferred technique, instead of the destructive technique of pulverizing and grinding the whole sample (Kolman and Tuross, 2000), as former technique retains the morphological features of the tooth for further forensic analyses (Brkic et al., 2000; O'Rourke et al., 2000). Although the speed and heat generated during drilling may degrade or denature the DNA structure (Lindahl, 1993), it has not been reported in human genetic forensic studies (Alakoc and Aka, 2009). The tooth type with the largest pulp chamber (i.e., molar) is the best source of DNA (De Leo et al., 2000). The amount and integrity of recovered DNA are considered a more critical issue when dealing with forensic archaeological teeth samples (Krings et al., 1997). Most ancient DNA studies (archaeological and forensic) to date have not utilized the most suitable skeletal material, namely, cementum-rich material at the tip of the tooth root, and have instead focused on dentine. Therefore, here we extracted DNA from cementum-rich material of 50 good-quality random sepoy teeth samples and analyzed mtDNA markers.

### 1.1 Historical Background of the Samples

**FIGURE 1:**
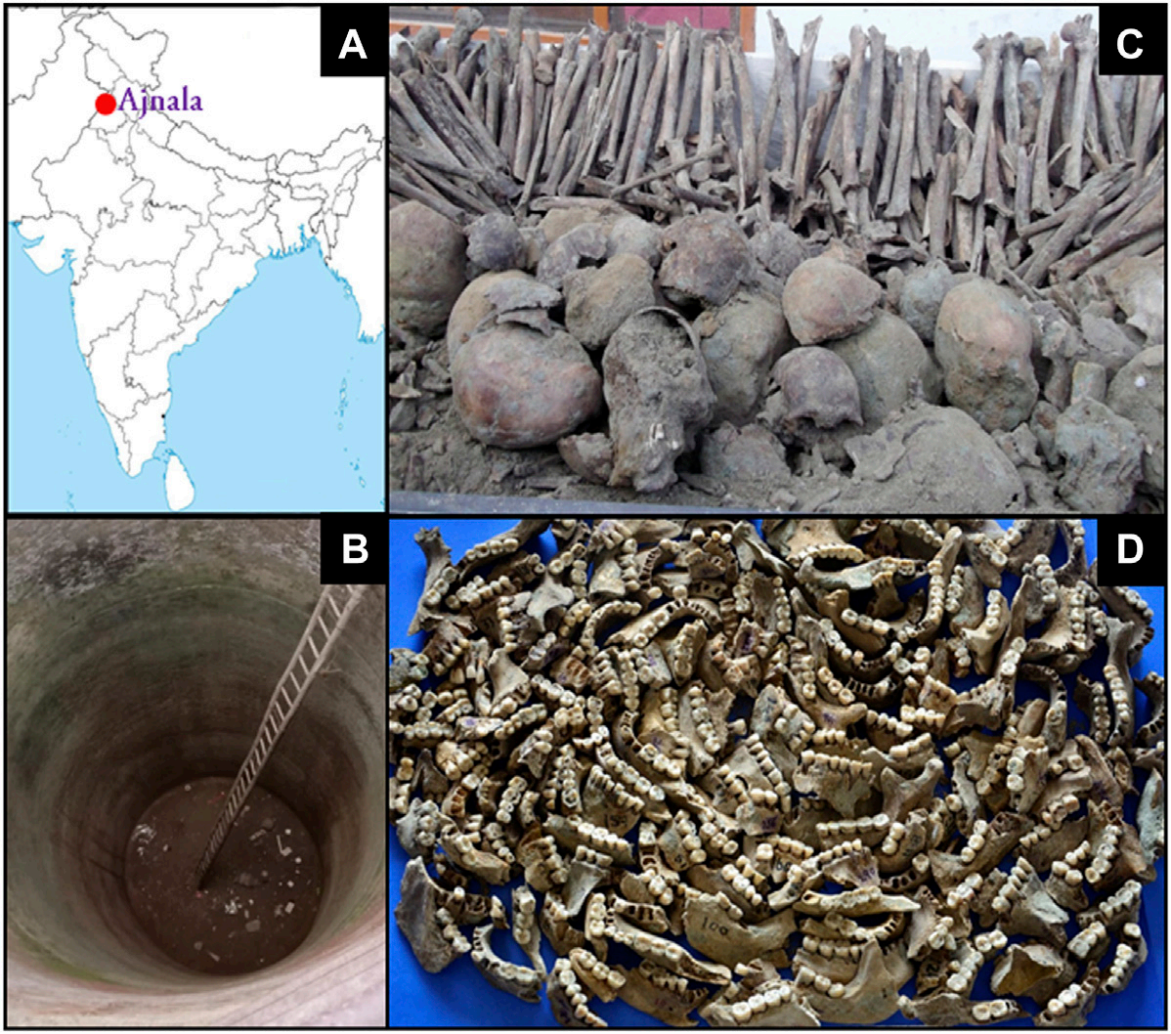
**(A)** Map of India showing study site Ajnala (red circle) from which the sepoy’s skeletal remains were excavated. **(B)** Abandoned deep well where a large number of skeletons were found. **(C)** Skulls and long bones collected from the abandoned well. **(D)** Mandibles with attached tooth samples.

The discovery of human remains and artifacts from the past is akin to chancing upon a goldmine. It presents an opportunity to understand the past with some degree of certainty and clarity. The human remains bring new knowledge concerning the biological, cultural, historical, and geographical realities of man in the bygone era. An obscure textbook authored by a civil servant of the British East India Company and Deputy Commissioner of Amritsar in 1857 (Cooper, 1858; Crispin Bates, 2017) mentioned a mass burial site in an abandoned well lying underneath a religious structure at Ajnala (Amritsar, India) (Figure 1). It documented the capture, imprisonment, and eventual killings of 282 Indian soldiers of the 26th Native Bengal Infantry regiment of the British Indian army. In May 1857, the Indian soldiers revolted against the colonial ordeal for the enforced use of beef- and pork-greased cartridges which escalated to a larger scale and further spread to various cantonments. The written accounts mention that the 26th Native Bengal Infantry battalion stationed at the Mian-Meer cantonment had soldiers only from the Indian states of Bengal, Bihar, Uttar Pradesh (eastern), and some northeastern states. After killing some British officers, the mutineer soldiers fled from the cantonment, although 282 were captured near Ajnala, Punjab, and were killed, and their bodies were dumped in the nearby disused well. Sociopolitical sensitivity of the incident and the emergent sanitary concerns were cited as the most immediate reasons for their disposal burial in the said well at Ajnala (Cooper, 1858; East India Papers 1859; Bates and Carter, 2017). Unfortunately, the book reference did not receive the attention it deserved from the state authorities. However, in February 2014, some local amateur archaeologists and curiosity seekers took it on their own to unearth the said, reported remains. They did not employ any scientific excavation technique for their exhumation from the well sediments, and in the process, the brittle skeletal remains were severely damaged, fragmented, and commingled.

Thus, the non-scientific excavation led to the retrieval of a large number of human remains along with some contextual items of identity such as coins, medals, bracelets, and rings. Two major hypotheses surround the origin of these skeletal materials: one hypothesis believes the remains belonged to the rebel soldiers as indicated in Cooper’s book (*The Crisis in Punjab: From 10th of May until Fall of Delhi*), East India Papers (1859), and Punjab Gazetteers. Some historians have ascribed the remains to the unfortunate mass killings perpetrated during the Indo-Pak partition conflicts of 1947. However, this claim stands dismissed by the engravings of images of Queen Victoria and the year of make on each coin and medal retrieved from the Ajnala skeletal assemblage as none of them has markings beyond 1856. Such claims were also refuted by the radiocarbon dates measured from some teeth collagen samples. Forensic anthropological analyses of these remains were undertaken to test the aforesaid theories regarding their origin. The commingled nature of Ajnala skeletal remains, the haphazard and diverse positioning of the skeletons in soil sediments, their taphonomic degradations, and non-scientific excavation have complicated their identification endeavors. Non-expert-mediated exhumation by the amateur excavators has further compromised the integrity of the already fragile and brittle remains. The government authorities’ delayed intervention led to the loss of forensic evidence of utmost national importance. Only teeth, jaw fragments, vertebrae, phalanges, skulls, femurs, clavicles, and hand and foot bones were retrieved among the skeletal debris; no pelvis, complete femur, sternum, etc. could be recovered from the remains (Sehrawat and Kaur, 2016; Sehrawat and Sankhyan, 2021). Thus, most analyses are based on the teeth preserved in a better state. Nonetheless, the first author of this article, to whom the remains were handed over, has cited the number of people as 246 based on dental counts, which is very close to the figure of 282 claimed in the written accounts (Sehrawat and Kaur, 2016). Through various forensic anthropological techniques, attempts have been made to examine the biological profile of the remains, bringing to light their identity parameters like age, sex, geographical origin, and dental health status. The analysis of the dental wears, caries, and hypoplasia has enabled the researchers to conclude their dental hygiene status, occupation, and dietary habits.

The preliminary analyses have supported the hypothesis that the remains belong to the mid-19th century as they are all adult men with good dental hygiene, indicating their military affiliations. There is historical and cultural evidence to support the geographic and temporal affiliations of Ajnala skeletal remains, which need to be scrutinized genetically/scientifically. Therefore, the present molecular and isotopic analyses were conducted to determine the local or non-local status of the recovered human remains, and whether the remains belonged to the reported geographical regions/states mentioned in the written records or not?

## 2 Methods

As the anthropological and odontological examinations provided some conflicting clues about the identity of the remains in question (Sehrawat JS 2016), 50 randomly selected molar teeth were subjected to mtDNA analyses and 85 samples for stable C and O isotope analyses. More than 165-year deposition of human remains in well sediments would influence the potential molecular profiling of the Ajnala skeletal remains. The data generated from this study are expected to expand our understanding of martyr soldiers’ ancestry and population affinity.

### 2.1 Ancient DNA Extraction and Mitochondrial DNA Genotyping

All mitochondrial DNA-related work was performed following strict aDNA standards in the clean laboratory of Birbal Sahni Institute of Palaeosciences, Lucknow, India. The outer surface of each tooth was cleaned manually with bleach to remove possible contaminants during excavation and split into two pieces, crown and root, using a sterile Dremel drill. The dentine and pulp were drilled out of the root using a fine drill bit, and the fine white powder was collected in sterile 2-ml Eppendorf tubes. aDNA extraction was done using an extraction buffer having 10 M urea, 0.5 M EDTA, and 10 µl of proteinase K (20 mg/ml), followed by incubation at 37°C for 24 h. After spinning down each tube at 4,000 rpm for 5 min, the pellet containing the cellular debris was discarded, and the supernatant containing the DNA was transferred to a 4-ml Amicon filter (Sigma-Aldrich®). It was performed to concentrate the samples with DNA since the volume was >1 ml. We brought the volume down to 250 ul by spinning the samples through the filters for 2–5 min. The supernatant was transferred to a 2-ml Eppendorf tube containing 5X PB binding buffer, that is, 5X 250 = 1.25 ml (Qiagen®). The PB–sample mix (for each tube separately) was transferred to MinElute spin columns (Qiagen®) and spun at 7,000 rpm for 1 min, and the eluate was discarded. A measure of 710 µl of PE wash buffer (Qiagen®) was added to the filters and spun at 10,000 rpm for 1 min. After discarding the supernatant, ethanol was used to wash off the salts, and each tube was spun empty at 14,000 rpm for a minute to remove any trace ethanol, followed by discarding the collection tube. The filters were placed in fresh 1.5-ml Eppendorf tubes, and 45 µl of EB elution buffer (Qiagen®) was added to the center of each filter. This was followed by incubation at 37°C for 15 min. After spinning the tube at 14,000 rpm for 1 min, an additional 30 μl EB buffer was added to the same filter (to recover any leftover bound DNA molecules), incubated for another 10 min at 37°C, and spun as before. The filter was finally discarded. This was followed for each tube.

The mtDNA genotyping experiment was performed on Sequenom’s MassARRAY platform, a powerful and flexible method for assaying up to a few thousand markers and up to thousands of individuals. It is based on distinguishing allele-specific primer extension products by mass spectrometry (MALDI-TOF). Most stages of the experimental protocol reflect adaptations of established PCR protocols to multiplexing, which allows the simultaneous amplification and detection of multiple markers per reaction.

In total, 115 mtDNA-specific diagnostic variations were carefully chosen to assign each sample to a specific haplogroup. We used a globally published mtDNA database (van Oven and Kayser, 2009) to select the diagnostic variations across human mtDNA. All the 115 polymorphic variations were amplified in four different pools, and genotyping was done on Sequenom iPLEX assay via the MassARRAY 28 system (SEQUENOM, San Diego, CA). This technology requires minimal input DNA 29 (pictogram scale) and is compatible with degraded, small-sized amplicons. Using this system, we designed four panels of amplification and extension primers, targeting 115 diagnostic mtDNA sites (23, 36, 31, and 25 sites, respectively). We performed multiplex PCR and genotyping in accordance with the manufacturer’s instructions. We report the genotyping results in Supplementary Data for the 50 samples from which we successfully extracted ancient DNA. We assigned mitochondrial haplogroups manually based on the combination of mutations (Supplementary Table S1).

All the haplogroups identified among the analyzed samples were pooled, and these data were considered a single population. Principal component analysis (PCA) was performed using POPSTR, which is provided by H. Harpending (Ahn et al., 2018). The state-wise frequency of haplogroup was obtained from the published sources (Metspalu et al., 2004; Chaubey et al., 2007; Chaubey et al., 2008; Thangaraj et al., 2008; Chaubey et al., 2011; Tamang et al., 2018).

### 2.2 Stable Isotope Analysis

#### 2.2.1 Oxygen Isotope and Carbon

The dentine powder of the selected tooth samples was collected in vials using a Dremel bit, and the powdered samples were kept in airtight centrifuge tubes to prevent any contamination. The organic and inorganic fractions were separated using standardized techniques. Approximately ∼ 500 μg samples were put in 12-ml vials and kept at 72°C in a Gas Bench tray and then flushed with ultrapure helium gas (≥99.9995%) to remove all atmospheric gasses from the vials. Further 50–70 μl phosphoric acid (≥99%) was added to the vials. All the samples were kept at 72°C for 45 min to equilibrate and produce CO_2_ gas. The produced CO_2_ was introduced into the IRMS through the GC column to analyze their isotopic ratio, and the isotopic ratios of carbon (δ^13^C) and oxygen (δ^18^O) were obtained using the SSH correction (Santrock et al., 1985). The stable isotopic composition of tooth samples were measured at the Stable Isotope Facility at the University of California, Davis, West Sacramento (United States) using a gas source continuous flow isotope ratio mass spectrometer (CF-IRMS) through Gas Bench.

## 3 Results and Discussion

### 3.1 Mitochondrial DNA Analysis

We randomly selected 50 sepoy samples collected from Ajnala, Punjab, and considered them a single population. We classified them into their respective haplogroup based on their mutation combination (Supplementary Table S1). In the sepoy group, at least thirteen haplogroups have been observed. Majority of the samples fell in the macrohaplogroup M (0.7). Moreover, with the current methodology used, we could classify a large number of samples at the sub-haplogroup level (Supplementary Table S1, S2). The west Eurasian-specific haplogroups observed were HV0e (0.06), U3 (0.02), and U7 (0.02). Among the haplogroups observed, the most frequent haplogroup was M39 (0.24), followed by R32 (0.14). These haplogroups are autochthonous to South Asia (Sun et al., 2006; Chaubey et al., 2008; Chandrasekar et al., 2009). Haplogroup M39 is widespread in South Asia; however, R32 is sporadic with a substantial presence in Gangetic plain populations (Supplementary Table S2).

**FIGURE 2:**
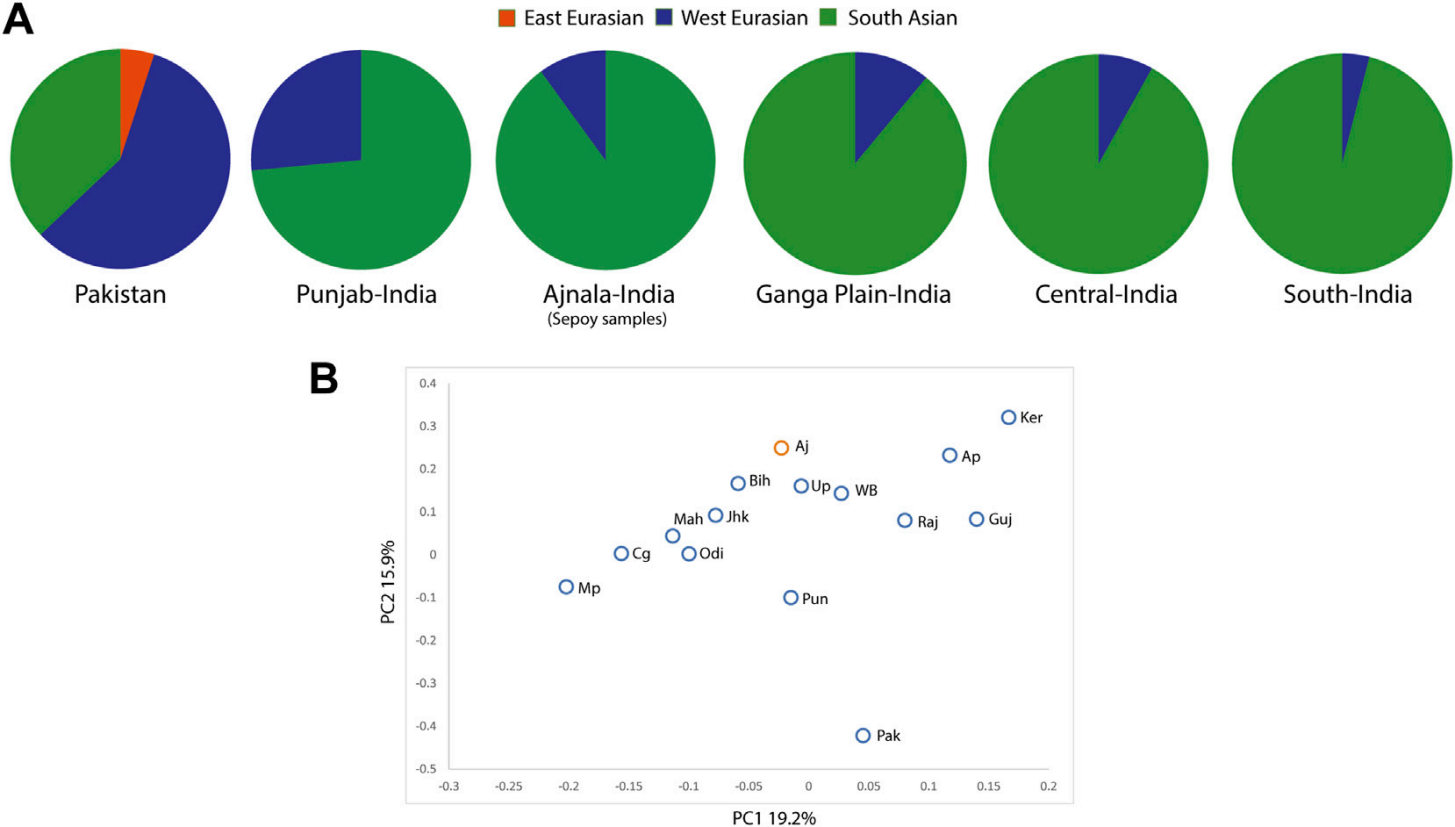
**(A)** Pie chart showing the proportion of the East Eurasian, West Eurasian, and South Asian genetic ancestries among the studied group (Ajnala, India), with respect to their adjacent geographical regions. South Asian haplogroups: M2-M6, M18, M25, M30-M67, N5, R5-R8, R30-R32, and U2a,b; west Eurasian haplogroups: HV, H, J, K, R0, R1, R2, U1-U5, U7, U8, U9, W, and X; east Eurasian haplogroups: A-G, M7-M12, R22, and N9. **(B)** Principal component analysis (PCA) on maternal haplogroups of South Asian populations, showing the affinity of sepoy samples with South Asian populations. Pak, Pakistan; Pun, Punjab India; Aj, Ajnala (sepoy samples); Raj, Rajasthan; Guj, Gujarat; Mah, Maharashtra; Up, Uttar Pradesh; Bih, Bihar; Jhk, Jharkhand; WB, West Bengal; Mp, Madhya Pradesh; Cg, Chhattisgarh; Odi, Odisha; Ap, Andhra Pradesh; Ker, Kerala.

It is well documented that the west Eurasian genetic component follows a clinal pattern in South Asia (Metspalu et al., 2004; Chaubey et al., 2007). Therefore, to pinpoint the genetic relatedness of the sepoy samples from Ajnala, Punjab, we first classified the observed haplogroups to their regional affiliations (Figure 2A). Populations native to Punjab and adjoining regions carried significantly higher West Eurasian maternal ancestry than the populations of Gangetic plain (two-tailed *p*-value < 0.005) (Figure 2A). The regional haplogroup spectrum of sepoy samples was more akin to the Gangetic plain populations, rather than local Punjabi populations. These results suggest their closer genetic affinity with the Gangetic plain populations, rather than the local Punjabi populations.

To better understand the genetic affiliation of our studied group, we have implemented principal component analysis (PCA) comparing its mtDNA profile with a dataset of haplogroup frequencies from Pakistani and Indian populations (Supplementary Table S2). We hypothesized that the placement of the sepoy samples in the PC plot would be closer to the states of their closest genetic similarity. We also observed a similar result in our PCA, where Indian populations were mainly distributed into two geographical clines. The sepoy group showed a genetic affinity with the Bihar, Uttar Pradesh, West Bengal, and Jharkhand state populations (Figure 2B). These observations placed their likely homeland near the north and east Indian geographic regions, mainly populated at the Gangetic plain.

### 3.2 Carbon and Oxygen Isotope

Isotopic studies have been widely used to examine diet and provenance in archaeological studies. The mean δ^18^O values for molar teeth samples analyzed in the present study are 24.5 ± 0.6‰ and 24.2 ± 1‰ (VSMOW), with values ranging from 23.3 to 25.8‰ (*n* = 42) and 21.4 to 26.0‰ (*n* = 46), respectively. Both the sites are characterized by the δ^18^O values larger than the range of ∼2‰. The δ^13^C values in the maxillary sites range from −12.5 to −4.6‰ and −13 to −1.7‰.

**FIGURE 3:**
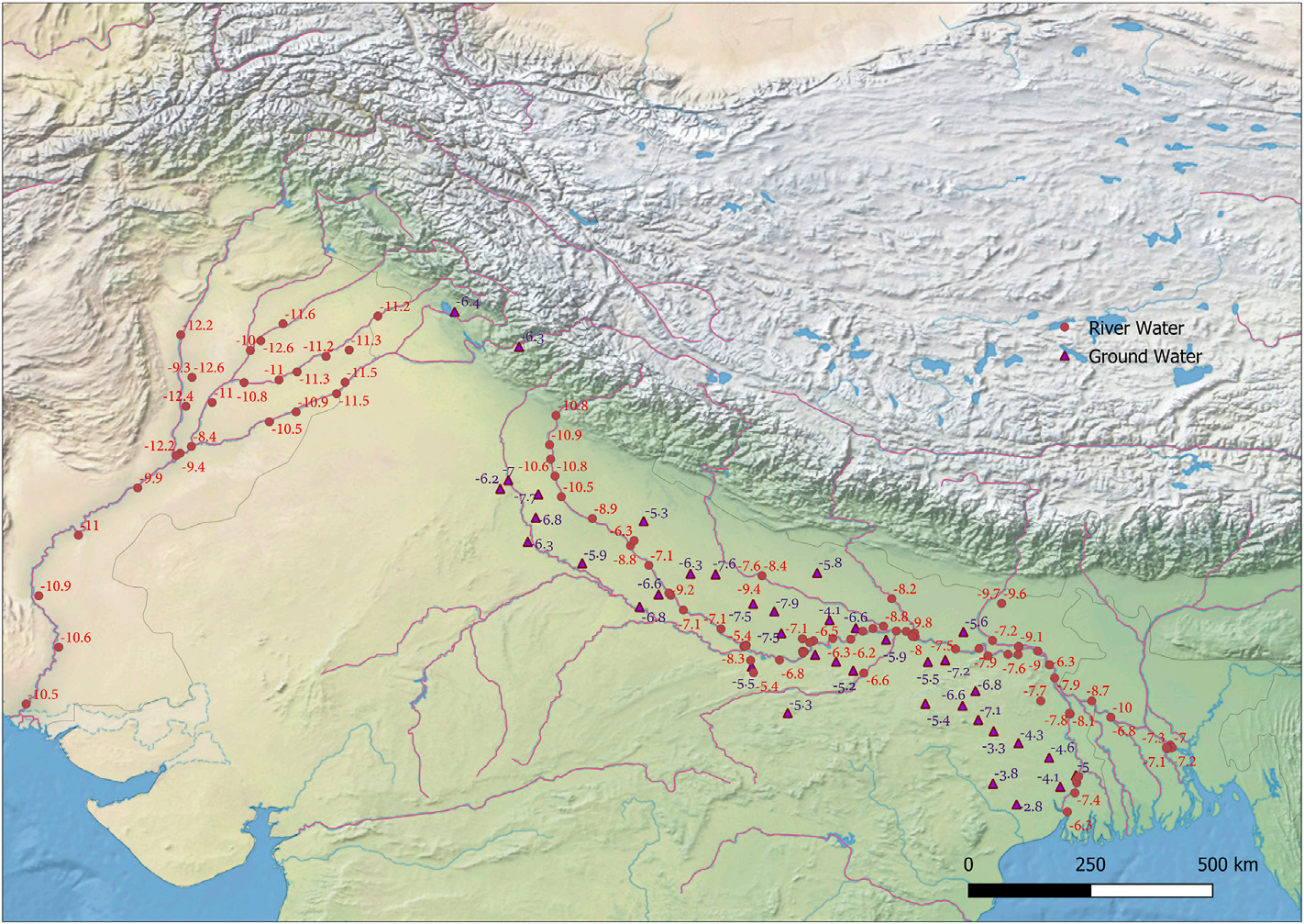
Map showing the spatial distribution of oxygen isotope ratio of Ganga and Indus River system (circles) and oxygen isotope ratios in groundwater (triangles) (Gupta et al., 2005; Karim, 2002).

The δ^18^O value in meteoric water varies regionally according to temperature and other climatic parameters, such as distance from the coastline, altitude, and latitude (Dansgaard, 1964; Rozanski et al., 2013). The δ^18^O values in the body are subject to several steps of metabolic fractionation. The fractionation mechanisms are relatively well known, allowing the calculation of approximate drinking water (δ^18^Ow) values from the δ^18^Oc of biogenic carbonate using conversion equations (Ehleringer et al., 2010; Posey, 2011). Although there are complexities in the calculation of meteoric water isotope composition in the past, the oxygen isotope composition of human remains has allowed for the identification of palaeomobility patterns. Oxygen isotope is usually used as a proxy for meteoric drinking water in mammals due to the direct correlation between δ^18^O of local meteoric drinking water, body water, and δ^18^O structural carbonate with subtle variations in fractionation according to species and climate variables (D’Angela and Longinelli, 1990; Daniel Bryant and Froelich, 1995; Daux et al., 2008; Kohn, 1996; Levinson et al., 1987; Longinelli, 1984; Luz and Kolodny, 1985; Luz et al., 1984). For this study, we used the equations of (Posey, 2011) (δ^18^Oc = 0.77*δ^18^Odw + 28.1‰) to calculate the δ^18^O values of drinking water. Using relationships between oxygen isotopes ratios of tooth carbonate (δ^18^Oc) and local precipitation (δ^18^Ow), precipitation δ^18^Ow values for the samples were calculated, which ranged from −3.0 to −6.2‰ to −2.7 to −8.8‰, respectively. Overall, the majority of δ^18^O values in the studied tooth samples fit “non-local people” to the excavation site and point to locations in the Gangetic plain (Uttar Pradesh) and coastal areas (like Orissa) (Figure 3). However, the lower δ^18^O values in the few samples suggest a small percentage of the local population (Figure 3).

The δ^13^C value of the carbonate associated with mammalian bioapatite is related to diet, is preserved on archaeological and geological time scales, and is widely used for reconstructing dietary preferences and availability of different food resources to mammals (Ambrose and Norr, 1993; Luz et al., 1990; Passey and Cerling, 2002; Passey et al., 2005a; Passey et al., 2005b; Stevens et al., 2006). It has been shown that the bioapatite carbonate is enriched in ^13^C by several per mil (9–15‰) relative to diet (Tieszen and Fagre, 1993; Cerling and Harris, 1999). Such enrichment in ^13^C is likely the result of fractionation during the precipitation of bioapatite minerals from dissolved inorganic carbon within the body. Therefore, the δ^13^C values of human tooth enamel reflect the diet, which varies with intake of relative proportion of C_3_ vs. C_4_ plant and intake of meat in the regional context.

The δ^13^C values of tooth samples range from −12.5 to −4.6‰ and −13 to −1.7‰, respectively, which, in turn, reveal dominant C_3_ to mixed C_3_–C_4_ diet patterns. Very high δ^13^C in two samples (HF-1 and F4) suggests a chief C_4_ diet. In the Indian context, the diet is rich in a mixture of C_3_ (legumes) and C_4_ (millets). The δ^13^C results from archaeo enamel samples from these two sites revealed that the dietary pattern matched with the modern human population from India with an admixture of C_3_ and C_4_.

Thus, in the present study, we could analyze DNA from human skeletal remains and stable isotope data to reconstruct the geographical origin of human skeletal remains excavated from an abandoned well for the first time. Analysis of two independent techniques (genetics and stable isotope) adds significant study novelty. Consequently, stable isotope and mtDNA analyses are consistent with the historical evidence, stating that the 26th Native Bengal Infantry battalion comprised Bengal, Odisha, Bihar, and eastern parts of Uttar Pradesh. Thus, the current research can uncover the hidden aspects of the struggle of the unknown martyrs against the colonial yoke. If reviewed in light of the findings of this study, the historical data and literature will further corroborate the incidence of this massacre and add another chapter in the annals of Indian history dedicated to the unsung heroes of India’s first freedom struggle. Our results expect to lay the groundwork and make a rich contribution to similar forensic investigations in the future for exploring the biological profile of remains even if recovered in mangled and damaged shape.

## Data Availability

The datasets presented in this study can be found in online repositories. The names of the repository/repositories and accession number(s) can be found in the article/Supplementary Material.
